# Trimanganese Tetroxide Nanozyme protects Cartilage against Degeneration by Reducing Oxidative Stress in Osteoarthritis

**DOI:** 10.1002/advs.202205859

**Published:** 2023-04-23

**Authors:** Wenhan Wang, Jiazhi Duan, Wenjun Ma, Bowei Xia, Feng Liu, Ying Kong, Boyan Li, Hang Zhao, Liang Wang, Keyi Li, Yiwei Li, Xiheng Lu, Zhichao Feng, Yuanhua Sang, Gang Li, Hao Xue, Jichuan Qiu, Hong Liu

**Affiliations:** ^1^ Department of Neurosurgery Qilu Hospital Cheeloo College of Medicine and Institute of Brain and Brain‐Inspired Science Shandong University Jinan 250012 P. R. China; ^2^ State Key Laboratory of Crystal Materials Shandong University Jinan 250100 P. R. China; ^3^ Shandong Key Laboratory of Brain Function Remodeling Jinan 250012 P. R. China; ^4^ Institute for Advanced Interdisciplinary Research (iAIR) University of Jinan Jinan 250022 P. R. China; ^5^ Department of Orthopedics Qilu Hospital Cheeloo College of Medicine Shandong University Jinan 250012 P. R. China

**Keywords:** cartilage degeneration, cartilage metabolism, osteoarthritis, trimanganese tetroxide nanozymes

## Abstract

Osteoarthritis, a chronic degenerative cartilage disease, is the leading cause of movement disorders among humans. Although the specific pathogenesis and associated mechanisms remain unclear, oxidative stress‐induced metabolic imbalance in chondrocytes plays a crucial role in the occurrence and development of osteoarthritis. In this study, a trimanganese tetroxide (Mn_3_O_4_) nanozyme with superoxide dismutase (SOD)‐like and catalase (CAT)‐like activities is designed to reduce oxidative stress‐induced damage and its therapeutic effect is investigated. In vitro, Mn_3_O_4_ nanozymes are confirmed to reprogram both the imbalance of metabolism in chondrocytes and the uncontrolled inflammatory response stimulated by hydrogen peroxide. In vivo, a cross‐linked chondroitin sulfate (CS) hydrogel is designed as a substrate for Mn_3_O_4_ nanozymes to treat osteoarthritis in mouse models. As a result, even in the early stage of OA (4 weeks), the therapeutic effect of the Mn_3_O_4_@CS hydrogel is observed in both cartilage metabolism and inflammation. Moreover, the Mn_3_O_4_@CS hydrogel maintained its therapeutic effects for at least 7 days, thus revealing a broad scope for future clinical applications. In conclusion, these results suggest that the Mn_3_O_4_@CS hydrogel is a potentially effective therapeutic treatment for osteoarthritis, and a novel therapeutic strategy for osteoarthritis based on nanozymes is proposed.

## Graphical abstract

Trimanganese tetroxide nanozyme with superoxide dismutase (SOD)‐like and catalase (CAT)‐like activities reduces oxidative stress‐induced damage in cartilage. Cross‐linked chondroitin sulfate carried with trimanganese tetroxide nanozyme performed well in osteoarthritis mice models. It presents a novel therapeutic strategy for osteoarthritis based on nanozymes.

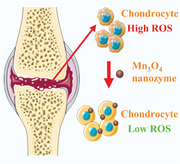

## 1 Introduction

Osteoarthritis (OA), a chronic degenerative cartilage disease characterized by joint pain and dysfunction, is the leading cause of movement disorders in humans.^[^ ^1^ ^]^ To date, more than 300 million people have been affected worldwide, which places a huge economic burden on society.^[^ ^2^ ^]^ A recent study reported that nearly 25% of adults are expected to suffer from OA by 2030 owing to the rapid growth of the aging population.^[^ ^3^ ^]^ Although OA has a high prevalence and disability, effective treatments for this widespread disease are lacking.^[^ ^4^ ^]^ Surgery is the only option for patients with late‐stage disease. For early‐stage patients, there is still no therapy to reverse or delay the progression of OA.^[^ ^5^ ^]^ Cartilage degradation, osteophyte formation, and chronic inflammatory reactions are the three typical pathological processes of OA.^[^ ^6^ ^]^ Although the pathogenic mechanism of OA remains unclear, oxidative stress‐induced inflammation has been shown to be a critical part of its occurrence and progression.^[^ ^7^ ^]^ It is widely believed that the cartilage is formed by chondrocytes and extracellular matrix (ECM).^[^ ^8^ ^]^ In the cartilage, chondrocytes generate constituents of the ECM, which, in turn, provides a survival environment for the chondrocytes; this positive feedback supports the metabolic balance in the cartilage.^[^ ^9^ ^]^ However, this positive feedback is disrupted by inflammation induced by oxidative stress because of an imbalance in the biomechanics of joints in the early stage of OA. Next, the expression of collagen and aggrecan, which are key constituents of ECM in the cartilage, is inhibited.^[^ ^10^ ^]^ Moreover, inflammatory cytokines and matrix hydrolases, such as interleukin‐1 (IL‐1), tumor necrosis factor *α* (TNF‐*α*), and matrix metalloproteinase (MMP13), which activate the inflammatory cascade to further exacerbate the progression of OA, are produced and released.^[^ ^11^ ^]^ Consequently, the initial handling of oxidative stress is the fundamental treatment to reverse the progression of OA.

Reactive oxygen species (ROS) produced in mammalian cells cause oxidative stress in OA chondrocytes.^[^ ^12^ ^]^ In chondrocytes, ROS levels are regulated by superoxide dismutase (SOD) and catalase (CAT), which convert ROS to molecular oxygen (O_2_) and water (H_2_O).^[^ ^13^ ^]^ This means that SOD and CAT form the first line to protect chondrocytes against oxidative damage; thus, we developed a new therapeutic strategy based on SOD and CAT activity. However, there are few SOD and CAT bioenzymes in the knee joint cartilage due to the uneven location of bioenzymes in the body, which limits the application of this therapy in OA. Moreover, owing to the rapid consumption of biological enzymes, frequent supplementation of biological enzymes is needed in the joints. Furthermore, supplemental biological enzymes can only be supplied through intraarticular injections, owing to the special structure of the knee joint. However, frequent intra‐joint injections of bioenzymes result in a huge physiological and psychological burden on patients.^[^ ^14^ ^]^ Additionally, application and storage are difficult because of the poor stability of bioenzymes, which further limits their use in OA. Thus, anti‐ROS therapy based on bioenzymes for OA treatment is almost impossible.

Fortunately, recent studies have shown that some nanomaterials that can reduce oxidation by denaturation in the cellular microenvironment act as bioenzymes to eliminate excessive intracellular ROS.^[^ ^15^ ^]^ Nanozyme, a type of nanomaterial that possesses intrinsic enzyme‐like activity, catalyzes enzyme substrates efficiently under mild conditions and exhibits catalytic efficiency and enzymatic reaction kinetics similar to those of natural enzymes.^[^ ^16^ ^]^ Compared with bioenzymes, nanozymes still maintain 85% catalytic activity even in strong acids/bases (pH ≈2–10) or over a wide temperature range (≈4–90 °C), which suggests broad clinical application prospects.^[^ ^16^, ^17^ ^]^ As described above, stable nanozymes may solve the problem of biological enzymes being easily inactivated and rapidly consumed. This indicates that application in OA may avoid frequent intra‐joint injections to remarkably reduce the physiological and psychological burden on patients. Recently, an increasing number of studies have reported that nanozymes are used in oncology therapy, Crohn's disease, hepatitis, brain injury, and others.^[^ ^18^ ^]^ Most recently, researchers have found that well‐designed nanozymes could act as SOD or CAT to regulate ROS levels, which enables an appropriate cue for ROS‐related OA therapy. Most nanozyme‐related studies focus on cancer therapy because of the ease of operation and availability of animal models.^[^ ^16^, ^17^, ^19^ ^]^ The application of nanozymes for OA therapy is in urgent demand, but a great challenge exists in interdisciplinary research between materials science and biomedical engineering. In recent years, Mn_3_O_4_ nanozymes have attracted considerable attention because of their highly enhanced SOD‐mimicking performance.^[^ ^20^ ^]^ As one of the essential trace elements in the human body, Mn has great biocompatibility, and its metabolite can also be used in other physical activities.^[^ ^21^ ^]^ In other words, the use of Mn_3_O_4_ nanozymes in treatment is safe with few side effects.^[^ ^22^ ^]^ Moreover, owing to the special structure of the knee joint, the use of nanozymes is challenging. The cartilage comprises a sparse population of chondrocytes within a dense ECM without blood or lymphatic vessels.^[^ ^23^ ^]^ When oxidative stress is experienced in the joint, the cartilage is vulnerable to oxidation and is a difficult target for drug delivery. Thus, in the current study, we designed a Mn_3_O_4_ nanozyme with a small particle size (6 nm) to rapidly reach the chondrocytes. Furthermore, compared with other nanozymes such as Fe_3_O_4_ and Fe_2_O_3_, the Mn_3_O_4_ nanozyme in this study had higher SOD activity. Compared with CeO_2_ and V_2_O_5_, the catabolite of Mn_3_O_4_ nanozymes can be used for other physiological activities of the body. Thus, the degradation and metabolism of Mn_3_O_4_ nanozymes have great advantages for OA treatment.

In joints, synovial vasculature and lymphatics are abundant and rapidly clear small molecules and large macromolecules.^[^ ^9^ ^]^ Thus, a better method to deliver Mn_3_O_4_ nanozymes is required to avoid elimination. To avoid frequent intraarticular injections and maintain lubricity in the joint, a crosslinked chondroitin sulfate (CS) hydrogel was used to carry Mn_3_O_4_ nanozymes through intraarticular injections. In this study, we proposed a strategy for OA therapy using Mn_3_O_4_ nanozymes. To mimic the OA environment, a human chondrocyte cell line (SW1353) was stimulated with hydrogen peroxide to simulate a high‐oxidative‐stress microenvironment. The SOD‐mimicking performance of the Mn_3_O_4_ nanozymes was assessed using this system in vitro. Moreover, crosslinked CS hydrogel was accepted in the current study as a carrier for Mn_3_O_4_ nanozymes and used in OA mouse models to assess the therapeutic effect of this treatment strategy. Collectively, this study presents the effects of Mn_3_O_4_ nanozymes in protecting chondrocytes against oxidative stress and proposes associated treatment strategies.

## 2 Results

### 2.1 Synthesis and Characterization of Mn_3_O_4_ Nanozyme

**Figure 1:**
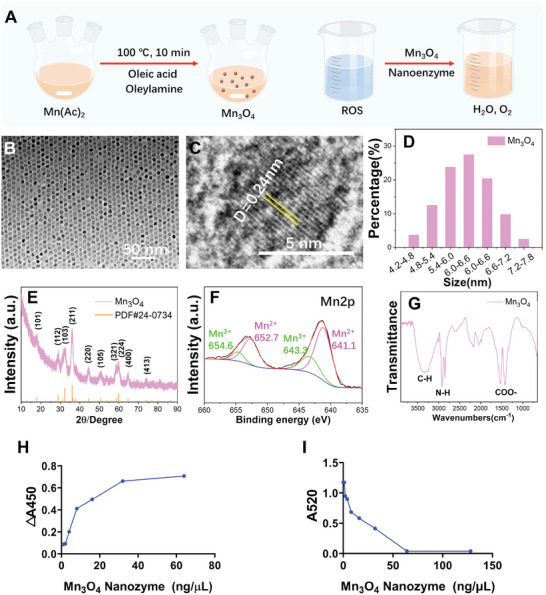
Characterization of synthesized Mn_3_O_4_ nanoparticles. A) Schematic of Mn_3_O_4_ nanozyme synthesis. B) TEM and C) HRTEM images of the Mn_3_O_4_ nanozyme. D) Size distribution, E) XRD pattern, F) XPS pattern, and G) FTIR pattern of the synthesized Mn_3_O_4_ nanozyme. H) SOD‐like activity of the Mn_3_O_4_ nanozyme (pH 7.4, 37 °C). I) CAT‐like activity of the Mn_3_O_4_ nanozyme (pH 7.4, 37 °C).

Mn_3_O_4_ nanozymes were synthesized using a chemical reaction method at 100 °C, and the synthesis process of Mn_3_O_4_ is shown in **Figure** **1**A (left). The SOD activity was checked by adding the nanozyme to a solution of oxygen free radicals, as shown in Figure 1A (right). The morphology of the obtained Mn_3_O_4_ nanoparticles was characterized using high‐resolution transmission electron microscopy (HRTEM). As shown in Figure 1B, the obtained nanoparticles were exceedingly uniform ultrasmall nanospheres of ≈6 nm in diameter. In addition, a TEM image of the Mn_3_O_4_ nanoparticles at low magnification (Figure S1, Supporting Information) confirmed the overall regularity of the synthesized nanoparticles. The HRTEM image (Figure 1C) analysis of the Mn_3_O_4_ nanoparticles confirmed that the obtained nanoparticles have good crystallinity, which endows the nanozyme with high reaction activity. The crystal plane spacing of 0.24 nm corresponds to 220 crystal planes. The size distribution of the obtained Mn_3_O_4_ nanoparticles was analyzed, as shown in Figure 1D. Statistical analysis of the nanoparticle diameters indicated that the nanoparticles were ≈4–8 nm, and the main size was 6 nm. The crystal structure of the obtained Mn_3_O_4_ nanoparticles, as characterized by X‐ray diffraction (XRD), is shown in Figure 1E. The results showed that the synthesized nanoparticles can be well indexed to the standard powder diffraction file card (PDF#24‐0734), indicating a pure phase of Mn_3_O_4_. X‐ray photoelectron spectroscopy (XPS) was used to characterize the valence states of the synthesized nanoparticles. As shown in Figure 1F, the spectrum of Mn_2p_ clearly shows peaks of Mn^2+^ and Mn^3+^, which confirms the composition of Mn_3_O_4_. The SOD and CAT activities of Mn_3_O_4_ nanozymes were also assayed in this study. The peaks in the Fourier transform infrared (FTIR) spectrum shown in Figure 1G located at 1532.6 and 1424.7 cm^−1^ indicate the existence of COO‐. The peaks located at 2921.8 and 2851.8 cm^−1^ indicate N–H bonds, and the broad peak located at 3363.4 cm^−1^ indicates C–H bonds. The FTIR results indicated some residual oleic acid and olamine molecules only on the surface of the Mn_3_O_4_ nanozymes without any other foreign substances. To further confirm the effect of residual oleic acid and olamine molecules on cells, a live/dead assay in SW1353 cells treated with Mn_3_O_4_ nanoparticles was performed. As shown in Figure S2, Supporting Information, the Mn_3_O_4_ nanoparticles produced in this study had great biocompatibility. To assess the SOD‐like activity of the Mn_3_O_4_ nanozymes, the SOD enzyme was used as a standard for further quantification in this study, and the associated results are displayed in Figure S3, Supporting Information. As shown in Figure 1H and Figure S3, Supporting Information, the Mn_3_O_4_ nanozymes had high SOD‐like activity. After the average calculation, the specific values of the SOD enzyme‐like activities were 120.00 U mg^−1^. Moreover, according to the kinetic curve of the enzymatic reaction (Figure S4A, Supporting Information), the activities of several concentrations (1–32 ng µl ^−1^) of Mn_3_O_4_ nanozymes at different time points were dose‐dependent. The SOD catalytic initial velocity of Mn_3_O_4_ nanozyme was detected according to the kinetics tests method under different concentrations of O^2−^. The reaction curve was shown in Figure S4B, Supporting Information. The results show that the *V*_max_ was 0.0486 × 10^7^ mol s^−1^ and the *K*_m_ was 0.017 mmol L^−1^. As described above, the validity of nanozymes was still high, even in strong acid/base or wide temperature ranges, which suggests broad clinical application prospects. Thus, the SOD activity of Mn_3_O_4_ nanozymes in a strong acid/base (pH = 4/10) and over a wide temperature range (4 and 80 °C) was tested in this study. As indicated in Figure S5, Supporting Information, the SOD enzyme presented almost no enzyme activity at a pH of 4 or 10, while the activity of Mn_3_O_4_ nanozymes was hardly affected. At the reaction temperatures of 4 and 80 °C, the activity of Mn_3_O_4_ nanozymes was still hardly affected, while the SOD enzyme showed nearly no enzyme activity (Figure S6, Supporting Information). In addition, the CAT activity of Mn_3_O_4_ nanozymes was tested in this study, and it was the same as that of the SOD enzyme. As shown in Figure 1I, the CAT enzyme and Mn_3_O_4_ nanozymes presented high CAT activity, and the specific values of the CAT enzyme‐like activities were 79.71 U mg^−1^. For CAT bioenzyme activity curves corresponding to nanozymes of different concentrations (Figure 1M), 128 ng µL^−1^ presented the same OD value as 64 ng µL^−1^, which indicated that the CAT activity of nanozymes (≥ 64 ng µL^−1^) exceeded the upper limit of kit detection. Moreover, the activity at several concentrations (1–32 ng µL^−1^) of Mn_3_O_4_ nanozymes at different time points was dose‐dependent, according to the kinetic curve of the enzymatic reaction (Figure S7A, Supporting Information). The CAT catalytic initial velocity of Mn_3_O_4_ nanozyme was detected according to the kinetics tests method under different concentrations of H_2_O_2_. The reaction curve was shown in Figure S7B, Supporting Information. The results show that *V*_max_ was 5.833 × 10^7^ mol s^−1^ and the *K*_m_ was 0.6 mmol L^−1^. In strong acids/bases (pH = 4/10) and under a wide temperature range (4 and 80 °C), the CAT activity of the Mn_3_O_4_ nanozymes was still maintained; meanwhile, the CAT enzyme was nearly inactivated, and its activity was the same as the SOD activity (Figures S8 and S9, Supporting Information). The Mn_3_O_4_ nanozyme presents stable SOD activity and CAT activity both in a normal reaction environment and under extreme reaction conditions, the main reason may be due to the higher stability of Mn_3_O_4_ nanozyme under these conditions compared with MnO_2_. In addition, the standard Gibbs free energy of formation (ΔfGm) of Mn2+ and Mn3+ is smaller than Mn4+ at acidic conditions and shows similar stability at alkalinity conditions, therefore, the Mn_3_O_4_ nanozyme shows stable activity under different PH. It is also an interesting study for the application of extreme conditions in the future. However, in this study, the application of Mn_3_O_4_ nanozyme was under physiological conditions. Collectively, Mn_3_O_4_ nanoparticles presented SOD and CAT activities in a normal reaction environment.

### 2.2 Mn_3_O_4_ Nanozymes protect against Oxidative Stress in SW1353 Cells

**Figure 2:**
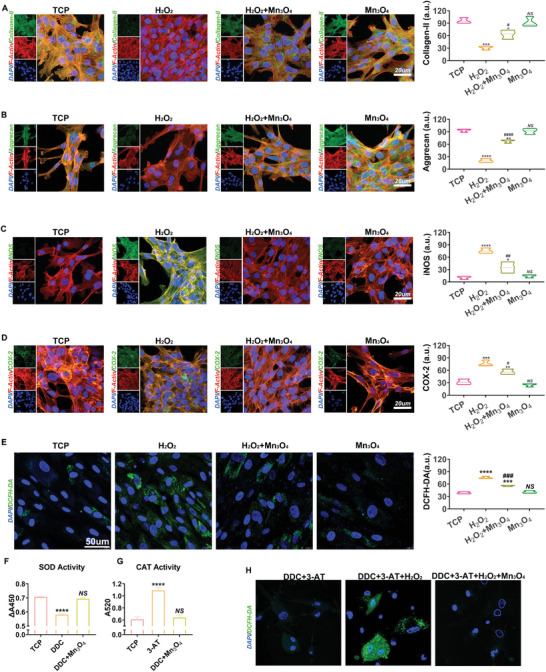
Mn_3_O_4_ nanozymes protected cartilage balance in chondrocytes. A) Expression level of collagen II was tested by immunofluorescent staining (*n* = 3, error bars indicate mean ± S.E.M). B) Aggrecan expression was tested by immunofluorescent staining (*n* = 3, error bars indicate mean ± S.E.M). C) Immunofluorescent staining of COX‐2 and associated expression level assay (*n* = 3, error bars indicate mean ± S.E.M). D) Immunofluorescent staining of iNOS and associated expression level assay (*n* = 3, error bars indicate mean ± S.E.M). E) ROS levels in the four groups assayed by DCFH‐DA (*n* = 3, error bars indicate mean ± S.E.M). F) SOD activity assay of cells after DDC or DDC + Mn_3_O_4_ treatment (*n* = 3, error bars indicate mean ± S.E.M). G) CAT activity assay of cells after 3‐AT or 3‐AT+ Mn_3_O_4_ treatment (*n* = 3, error bars indicate mean ± S.E.M). H) ROS level in cells after the treatment with DDC, 3‐AT, or H_2_O_2_ + Mn_3_O_4_ (*n* = 3, error bars indicate mean ± S.E.M).*p < 0.05 versus TCP group; **p < 0.01 versus TCP group; ***p < 0.001 versus TCP group; ****p < 0.0001 versus TCP group; # *p* < 0.05 versus H_2_O_2_ group; ## *p* < 0.01 versus H_2_O_2_ group; ### *p* < 0.001 versus H_2_O_2_ group; #### *p* < 0.001 versus H_2_O_2_ group; *NS*: not significant.

To assess the therapeutic effect of Mn_3_O_4_ nanozymes on chondrocytes, an inflammatory microenvironment induced by high levels of oxidative stress was created by adding H_2_O_2,_ and then treated with Mn_3_O_4_ nanozymes. Cells cultured in a tissue culture plate (TCP) without any treatment were used as negative controls. Collagen II, the main component of the cartilage matrix, is produced by normal chondrocytes to maintain cartilage matrix metabolism and protects the cartilage against degeneration in the development of OA. As shown in **Figure** **2**A, no difference in collagen II expression was observed between the TCP group and Mn_3_O_4_ nanozyme group, which suggests that Mn_3_O_4_ nanozymes do not inherently affect the expression of collagen II. However, comparing the TCP group, H_2_O_2_‐treated group, and H_2_O_2_ and Mn_3_O_4_ nanozyme group, the expression of collagen II was significantly downregulated by a factor of two after stimulation with H_2_O_2_ but was rescued by Mn_3_O_4_ nanozyme treatment. The results shown in Figure 2A indicate that Mn_3_O_4_ nanozymes rescued the expression of collagen II in chondrocytes under oxidative stress. Aggrecan, a proteoglycan originally isolated from cartilage, is another component of the cartilage matrix produced by normal chondrocytes. The normal supply of aggrecan maintains the balance of cartilage tissue metabolism and reduces the inflammation caused by joint instability. Figure 2B displays the differential expression of aggrecan in all groups. Similar to collagen II, Mn_3_O_4_ nanozymes did not inherently affect the expression of aggrecan in the TCP and Mn_3_O_4_ nanozyme groups. A nearly threefold decrease in aggrecan expression was observed after H_2_O_2_ treatment and was rescued by Mn_3_O_4_ nanozyme treatment. Decreased aggrecan levels in the H_2_O_2_‐treated group revealed that H_2_O_2_‐induced high ROS levels obstructed the normal expression of aggrecan in chondrocytes, leading to cartilage metabolic disorders. However, the addition of Mn_3_O_4_ nanozymes restored the function of chondrocytes, which was confirmed by comparing the H_2_O_2_‐treated group with the H_2_O_2_‐and Mn_3_O_4_ nanozyme‐treated groups. In summary, these results revealed that the function of chondrocytes was destroyed by H_2_O_2_ and restored by Mn_3_O_4_ nanozymes.

Oxidative stress‐induced inflammation in chondrocytes was assessed in this study. Inducible nitric oxide synthase (iNOS) mediates the expression of inflammatory cytokines, suppresses the synthesis of collagen and aggrecan, and induces chondrocyte cell death.^[^ ^24^ ^]^ In chondrocytes, iNOS is activated and released by oxidative stress‐induced inflammation, whereas iNOS contributes to oxidative stress by producing more oxidative agents. As shown in Figure 2C, no significant difference was found between the TCP and Mn_3_O_4_ nanozyme groups, indicating that the addition of Mn_3_O_4_ nanozymes did not interfere with the normal function of chondrocytes. A nearly threefold increase in the expression of iNOS was found between the TCP and H_2_O_2_‐treated groups, which indicates that high iNOS levels are induced by oxidative stress. Next, the increased iNOS levels were repressed by the addition of Mn_3_O_4_ nanozymes. In addition to iNOS, cyclooxygenase 2 (COX2), which is involved in the antioxidant defense system under oxidative stress, is activated in chondrocytes after H_2_O_2_ stimulation. After activation by oxidative stress, the iNOS‐induced COX2‐mediated pathway further disturbs the balance between oxidative stimulators and inhibitors, leading to an inflammatory reaction.^[^ ^25^ ^]^ The results, as shown in Figure 2D, indicated that H_2_O_2_ treatment successfully activated the expression of COX2, which was in line with the expression of iNOS. Treatment with Mn_3_O_4_ nanozymes protected chondrocytes against oxidative stress‐induced inflammation. To assess the anti‐ROS effect of the Mn_3_O_4_ nanozymes, a DCFH‐DA fluorescent probe was used to test the level of cellular ROS. As shown in Figure 2E, H_2_O_2_ treatment significantly increased the level of ROS, and Mn_3_O_4_ nanozymes inhibited this increase. Moreover, a superoxide assay further confirmed that H_2_O_2_ treatment successfully destroyed the mitochondrial respiratory chain, and Mn_3_O_4_ nanozymes effectively reduced superoxide accumulation (Figure S10, Supporting Information). To further verify the SOD‐like or CAT‐like activities of Mn_3_O_4_ nanozymes, sodium diethyldithiocarbamate (DDC, SOD inhibitor) and 3‐amino‐1,2,4‐triazole (3‐AT, CAT inhibitor) were used to inhibit the expression of intracellular SOD and CAT biological enzymes. As shown in Figure 2F,G, SOD and CAT activities were significantly inhibited by DDC and CAT, while treatment with Mn_3_O_4_ nanozymes increased intracellular SOD and CAT activities, respectively. In addition, after DDC+3‐AT treatment, ROS levels were increased by H_2_O_2_ but suppressed by Mn_3_O_4_ nanozymes (Figure 2H). Collectively, these results reveal that Mn_3_O_4_ nanozymes inhibited the inflammatory reaction stimulated by ROS and disrupted the vicious cycle created by oxidation and inflammation through SOD‐like and CAT‐like activities.

**Figure 3:**
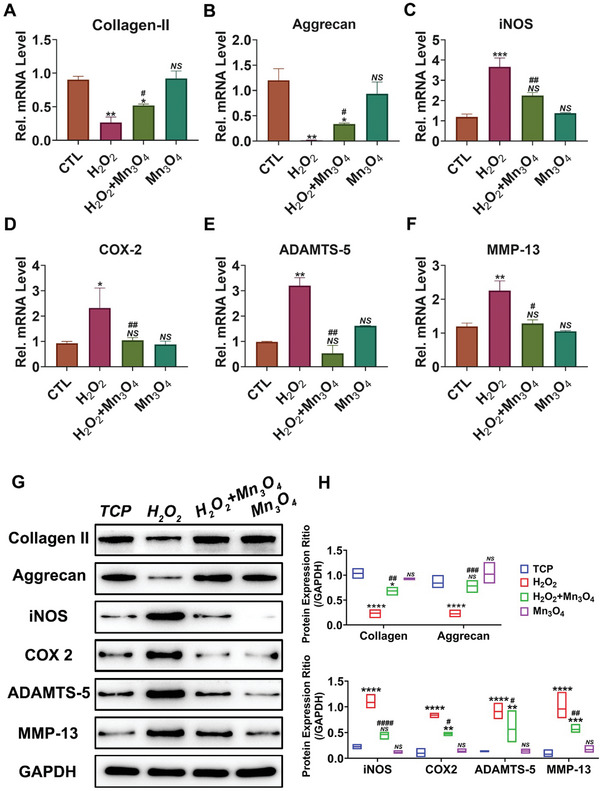
Mn_3_O_4_ nanozymes protect cartilage against degeneration in vitro. mRNA levels of A) collagen II, B) aggrecan, C) COX‐2, D) iNOS, E) ADAMTS‐5, and F) MMP13 were determined by real‐time PCR (*n* = 3, error bars indicate mean ± S.E.M). G) Protein levels in each group were assayed by western blotting, and H) the expression ratio was also assayed (*n* = 3, error bars indicate mean ± S.E.M). *p < 0.05 versus TCP group; **p < 0.01 versus TCP group; ***p < 0.001 versus TCP group; ****p < 0.0001 versus TCP group; # *p* < 0.05 versus H_2_O_2_ group; ## *p* < 0.01 versus H_2_O_2_ group; ### *p* < 0.001 versus H_2_O_2_ group; #### *p* < 0.001 versus H_2_O_2_ group; *NS*: not significant.

To further clarify the associated mechanism of Mn_3_O_4_ nanozymes in OA treatment, the expression of matrix proteins and associated inflammatory factors was quantified in both gene transcription and protein translation. Because collagen II and aggrecan are the main components of the cartilage matrix, their production determines the level of cartilage degeneration to some extent. **Figure** **3**A indicates that the mRNA level of collagen II was repressed to nearly 30% by H_2_O_2_ treatment, and this repression was reversed by Mn_3_O_4_ nanozyme treatment. The mRNA level of aggrecan was decreased to 10% after H_2_O_2_ treatment but was rescued by Mn_3_O_4_ nanozyme treatment (Figure 3B). iNOS and COX‐2 are inflammatory biomarkers that reflect the extent of the inflammatory response. Mn_3_O_4_ nanozymes successfully reduced the mRNA levels of iNOS and COX‐2 (Figure 3C,D), which were increased after H_2_O_2_ treatment. These results confirmed that Mn_3_O_4_ nanozymes protected chondrocytes against oxidative stress‐associated inflammation and maintain normal metabolism. In cartilage tissue degeneration, the production rate of the matrix from chondrocytes is lower than the hydrolysis rate. The production rate of the matrix depends on the function of chondrocytes, whereas the hydrolysis rate of the matrix depends on the content of various hydrolases.^[^ ^26^ ^]^ A disintegrin and metalloproteinase with thrombospondin motifs (ADAMTS‐5), also called aggrecanase 5, which maintains cartilage matrix balance by degrading aggrecan, shows abnormally high expression during the progression of OA.^[^ ^27^ ^]^ As indicated in Figure 3E, the stimulation of H_2_O_2_ remarkably activated the transcript level of ADAMTS‐5, which further reduced the content of aggrecan through proteolysis. Mn_3_O_4_ nanozymes inhibited the increased mRNA levels of ADAMTS‐5 stimulated by H_2_O_2_, which demonstrates that Mn_3_O_4_ nanozymes maintain the balance of cartilage tissue by slowing the hydrolysis rate of aggrecan. Matrix metalloproteinase 13 (MMP13), also called collagenase 3, plays a crucial role in cartilage destruction by hydrolyzing collagen.^[^ ^23^ ^]^ In the cartilage matrix, MMP13 and ADAMTS‐5 participate in the balance between hydrolysis and the production of matrix proteins, which form the matrix microenvironment. A significant result was that the mRNA level of MMP13 increased after stimulation with H_2_O_2_, but this increase was repressed by Mn_3_O_4_ nanozyme treatment (Figure 3F). That is, the transcription of MMP13 was in line with that of ADAMTS‐5, both of which contribute to the hydrolysis of cartilage, whereas Mn_3_O_4_ nanozymes inhibit it. Proteins are functional substances in the body of organisms and successful gene transcription does not mean that proteins are expressed. Therefore, the gene transcription and protein expression of cartilage degeneration‐associated biomarkers were assayed in this study. As shown in Figure 3G,H, the expression levels of collagen II and aggrecan in all groups were aligned with their mRNA levels, which further confirmed the therapeutic effects of Mn_3_O_4_ nanozymes on matrix expression. Moreover, the increased inflammatory levels induced by H_2_O_2_ were repressed by Mn_3_O_4_ nanozyme treatment, as shown by the expression levels of iNOS and COX‐2 (Figure 3G,H). Furthermore, the expression levels of matrix hydrolases such as ADAMTS‐5 and MMP13 were measured. Mn_3_O_4_ nanozymes successfully decreased the high expression levels of ADAMTS‐5 and MMP13 caused by H_2_O_2_ (Figure 3G,H). In summary, because of oxidative stress and inflammation, the hydrolysis rate of cartilage tissue was accelerated and matrix production was suppressed, which was reversed by Mn_3_O_4_ nanozyme treatment by inhibiting the hydrolysis of cartilage matrix and promoting the expression of associated proteins to supply the cartilage matrix.

In the pathological process of OA, due to oxidative stress and inflammatory pressure, the matrix hydrolysis progress accelerated, while the matrix production progress slowed, indicating that the cartilage metabolism balance was destroyed. As chondrocytes are the core of the cartilage tissue, their normal function determines the balance of cartilage metabolism to some extent.^[^ ^24^, ^28^ ^]^ In this study, the normal function of chondrocytes was destroyed by H_2_O_2_ treatment but was rescued by Mn_3_O_4_ nanozymes, suggesting that Mn_3_O_4_ nanozymes may have a good therapeutic effect in OA treatment. Although the in vitro therapeutic effect has been explored, the therapeutic effect in vivo still needs to be evaluated. Moreover, because of the poor blood supply in the cartilage, systemic administration of Mn_3_O_4_ nanozymes is limited in OA treatment. To further enhance the utilization rate of Mn_3_O_4_ nanozymes, systemic administration was abandoned, and intraarticular injections were performed. In the current study, we report a therapeutic strategy for OA based on Mn_3_O_4_ nanozymes through Mn_3_O_4_ @CS hydrogel intraarticular administration. In this therapeutic strategy, cross‐linked CS hydrogel was used as a nanozyme carrier to maintain lubrication in the articular cavity, and its decomposition product has been reported to reduce pain from OA.^[^ ^29^ ^]^ To test the toxicity of the CS hydrogel and Mn_3_O_4_ @CS hydrogel, a Cell Live/Dead Assay was performed. As shown in Figure S11, Supporting Information, the CS and Mn_3_O_4_ @CS hydrogels exhibited great biocompatibility.

### 2.3 Mn_3_O_4_ @CS Hydrogel protects Cartilage against Degeneration in Mice

**Figure 4:**
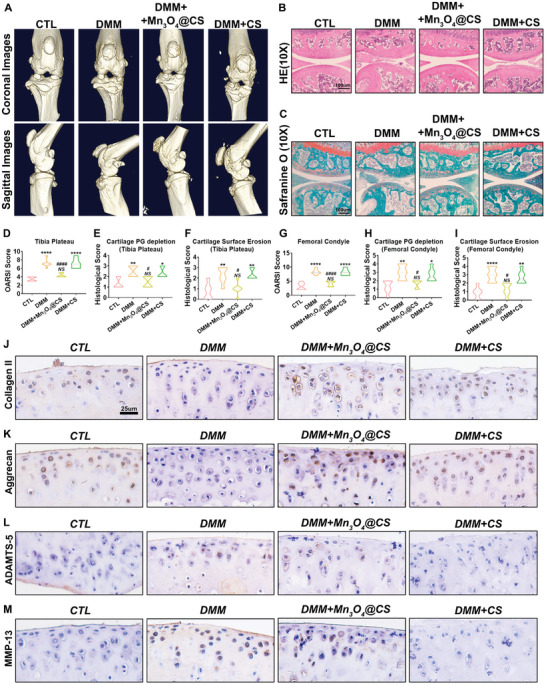
Mn_3_O_4_@CS hydrogel protected cartilage against degeneration in mice. A) Representative micro‐CT images of each group. Cartilage degeneration was observed in all groups, as assayed by B) HE and C) safranin O staining. D)OARSI score of the tibial plateau, E) cartilage PG depletion score of the tibial plateau, F) cartilage surface erosion score of the tibial plateau, G) OARSI score of the femoral condyle, H) cartilage PG depletion score of the femoral condyle, and I) cartilage surface erosion score of the femoral condyle (*n* = 3; error bars indicate mean ± S.E.M). J) Protein expression levels of collagen II, K) aggrecan, L) ADAMTS‐5, and M) MMP‐13 expression in the cartilage of the knee in all groups were tested by immunostaining. *p < 0.05 versus CTL group; **p < 0.01 versus CTL group; ***p < 0.001 versus CTL group; ****p < 0.0001 versus CTL group; # *p* < 0.05 versus DMM group; ## *p* < 0.01 versus DMM group; ### *p* < 0.001 versus DMM group; #### *p* < 0.001 versus DMM group; NS: not significant.

To measure the therapeutic effect of the Mn_3_O_4_@CS hydrogel in OA, a destabilization of the medial meniscus (DMM) mouse model was established, and Mn_3_O_4_@CS hydrogel treatment was performed. As shown in **Figure** **4**A, the representative micro‐computed tomography (micro‐CT) image analysis of the DMM group indicated that DMM surgery successfully caused OA in the knee joint, in which the articular surface was destroyed with many osteophytes. Fortunately, in the Mn_3_O_4_@CS hydrogel treatment group, the degree of damage was significantly improved, while the improvement in the CS hydrogel treatment group was fairly limited, which confirmed the therapeutic effect of the Mn_3_O_4_@CS hydrogel treatment. After protein fixation with paraformaldehyde, 5‐µm‐thick paraffin tissue slices were prepared, and HE staining was performed to assay cartilage disruption and associated inflammatory changes. As shown in Figure 4B, compared with the DMM group, Mn_3_O_4_@CS hydrogel treatment markedly reduced cartilage disruption and inflammatory cell recruitment caused by DMM surgery, whereas the therapeutic effects for the CS hydrogel treatment group were not evident. To further assess the degree of cartilage damage, safranin O/fast green staining was performed. In this staining, owing to the difference between bone and cartilage tissues, the cartilage tissue was dyed red by safranin‐O, while the bone tissue was dyed green by fast green. As shown in Figure 4C, a thin and destroyed cartilage surface was observed in the DMM‐ and CS hydrogel‐treated groups. However, the thickness of the cartilage and completeness of the cartilage surface were maintained after the Mn_3_O_4_@CS hydrogel injection. To further quantify the degree of cartilage damage, the OARSI and histological scores of both the tibial plateau and femoral condyle in all groups were determined. As shown in Figure 4D,G, the increase in the OARSI score in the DMM group indicates the success of the OA model establishment, whereas the decrease indicates the therapeutic effect of Mn_3_O_4_@CS treatment. Similar results were observed for cartilage PG depletion and cartilage surface erosion score (Figure 4E,F,H,I). In conclusion, Mn_3_O_4_@CS hydrogel therapy performed well in the treatment of OA in mice, as demonstrated by the morphological and histological results. Moreover, the balance between cartilage matrix production and hydrolyzation was also assessed in vivo by measuring the expression of the matrix and associated hydrolases. As shown in Figure 4J–M, the contents of collagen II and aggrecan, which are the main components of the cartilage matrix, were decreased after DMM surgery and rescued by Mn_3_O_4_@CS hydrogel treatment. Correspondingly, as shown in Figure 4J–M, ADAMTS‐5 and MMP13 contents were increased by DMM surgery, while this tendency was repressed by Mn_3_O_4_@CS hydrogel treatment.

**Figure 5:**
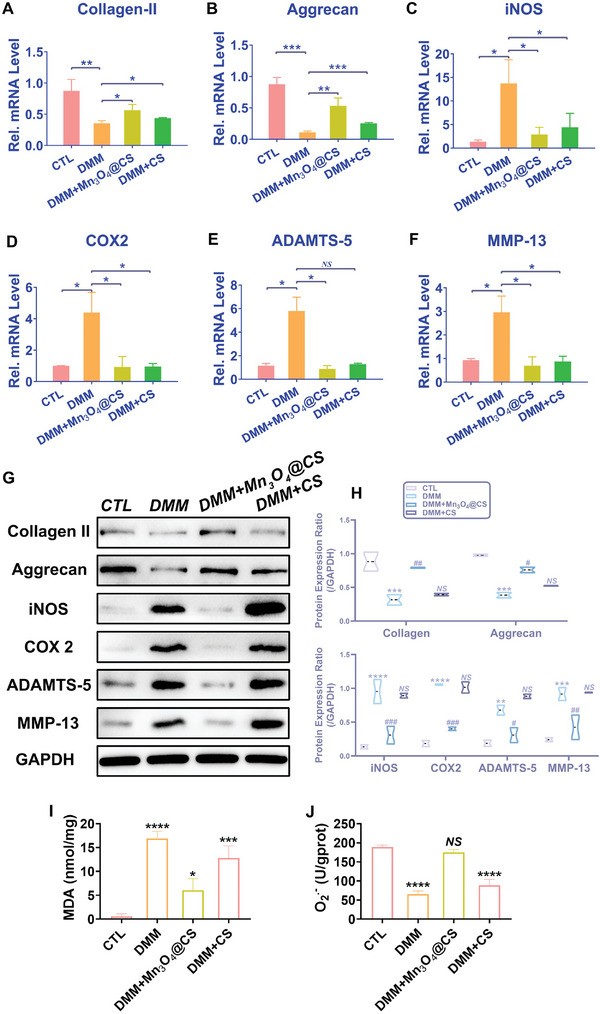
Mn_3_O_4_@CS hydrogel suppressed cartilage degeneration caused by DMM surgery in mice. mRNA levels of A) collagen II, B) aggrecan, C) iNOS, D) COX‐2, E) ADAMTS‐5, and F) MMP13 in the cartilage of the knee of each group were tested by real‐time PCR (*n* = 3, error bars indicate mean ± S.E.M). Protein expression levels of collagen II, aggrecan, iNOS, COX2, ADAMTS‐5, and MMP‐13 in the cartilage of the knee in all groups were tested by G) western blotting, and H) the expression ratio was also assayed (*n* = 3, error bars indicate mean ± S.E.M). I) MDA assay of the knee joint for the four groups (*n* = 3, error bars indicate the mean ± S.E.M). J) Superoxide anion assay of the knee joint for the four groups (*n* = 3, error bars indicate the mean ± S.E.M). *p < 0.05 versus CTL group; **p < 0.01 versus CTL group; ***p < 0.001 versus CTL group; ****p < 0.0001 versus CTL group; # *p* < 0.05 versus DMM group; ## *p* < 0.01 versus DMM group; ### *p* < 0.001 versus DMM group; #### *p* < 0.001 versus DMM group; NS: not significant.

To further quantify the differences in expression in these groups, total mRNA was extracted from each joint, followed by qPCR. An inspection of **Figure** **5**A,B further revealed that the mRNA levels of collagen II and aggrecan were reduced to 35% and 15% in the DMM group and upregulated to 70% and 65% in the Mn_3_O_4_@CS‐treated group, respectively. This finding indicates that the production of collagen II and aggrecan was inhibited by DMM surgery, but was rescued by Mn_3_O_4_@CS hydrogel treatment. For matrix hydrolase, the mRNA levels of ADAMTS‐5 and MMP13 were increased by DMM surgery but were repressed by Mn_3_O_4_@CS hydrogel treatment, which is similar to the immunohistochemical results. Notably, Figure 5A,B shows that the increased inflammatory level in the DMM group was also repressed by Mn_3_O_4_@CS hydrogel administration, which was investigated by measuring the expression levels of iNOS and COX2. These results confirm that the Mn_3_O_4_@CS hydrogel recovers the balance between cartilage matrix production and hydrolyzation, which was destroyed during the progression of OA. To further investigate the expression of these biomarkers at the protein level, the total protein from each group was extracted and subjected to western blotting. As shown in Figure 5C,D, the mRNA levels of collagen II and aggrecan were inhibited by DMM surgery but were recovered by the Mn_3_O_4_@CS hydrogel, which was consistent with the gene transcription level. The tendencies of iNOS, COX2, ADAMTS‐5, and MMP13 expression were also consistent with these mRNA levels, as described above, which further confirms the therapeutic effect of the Mn_3_O_4_@CS hydrogel in OA treatment and further explores the associated mechanism. To further investigate ROS levels in vivo, malondialdehyde (MDA) assay and superoxide anion assays were performed as previously reported.^[^ ^30^ ^]^ As shown in Figure 5I,J, the ROS level was remarkably increased in the DMM model and suppressed by Mn_3_O_4_@CS hydrogel treatment. The inhibition ability of superoxide anions was also elevated after Mn_3_O_4_@CS hydrogel treatment of the knee joint. Collectively, as a well‐accepted mouse model, the DMM mouse model, which displayed high similarity with the normal progression of human OA, provides an excellent model to assess the effect of Mn_3_O_4_ in vivo. As described above, the inflammation and imbalance in cartilage metabolism caused by DMM surgery were successfully rescued by Mn_3_O_4_@CS hydrogel treatment.

## 3 Conclusion

**Figure 6:**
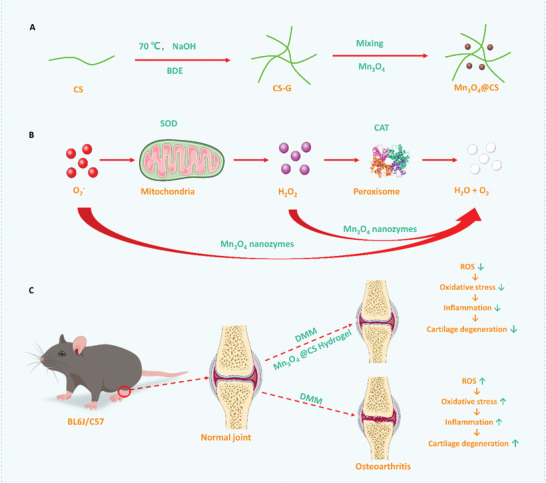
Graphical abstract of the Mn3O4 nanozyme‐based therapeutic strategy for osteoarthritis.

In summary, we demonstrated the protective effect of Mn_3_O_4_ nanozymes on chondrocytes during the progression of cartilage degeneration (**Figure** **6**). An investigation of the mechanism revealed that Mn_3_O_4_ nanozymes decreased ROS levels in chondrocytes by simulating SOD and CAT activity, which reduced the damage caused by high oxidative stress status in chondrocytes, and thus inhibited cartilage degeneration during the progression of OA. Furthermore, inflammation induced by chondrocyte dysfunction was also inhibited by the SOD and CAT effects of Mn_3_O_4_ nanozymes, which disrupted the positive feedback loop whereby chondrocyte dysfunction led to inflammation and inflammation enhanced chondrocyte dysfunction in return. We proposed a strategy for inhibiting OA development through cartilage protection using Mn_3_O_4_ nanozymes. This suggests that the Mn_3_O_4_ nanozyme‐based therapeutic strategy for OA may provide a new therapeutic strategy for patients with OA.

## 4 Experimental Section

### Materials

Manganese (II) acetate tetrahydrate, 1‐octadecene, oleylamine, oleic acid, sodium hydroxide, chondroitin 4‐sulfate sodium salt (CS), and 1,4‐butanediol diglycidyl ether were purchased from Shanghai Macklin Biochemical Co. Ltd. (Shanghai). Acetone, n‐hexane, and hydrochloric acid were purchased from Sinopharm Chemical Reagent Co. Ltd. All the chemicals were used without further purification.

### Synthesis of Mn_3_O_4 N_anoparticles, CS Hydrogel, and Mn_3_O_4_@CS

Mn_3_O_4_ nanozymes were synthesized by the thermal decomposition method, as described in a previous report.^[^ ^31^ ^]^ Briefly, 0.49 g manganese (II) acetate tetrahydrate was added to a mixed solution of 1.14 g oleic acid, 6.05 g oleylamine, and 30 mL 1‐octadecene. The mixture was stirred for 4 h at room temperature, and then slowly heated to 100 °C. Then, 0.38 mL of 0.05 M manganese (II) acetate tetrahydrate aqueous solution was added to the mixed solution before the heating temperature reached 100 °C. The reaction was allowed to proceed for 10 min and then cooled to room temperature. Mn_3_O_4_ nanozymes were obtained by adding a mixture of acetone and n‐Hex.

CS hydrogel was obtained by a crosslinking reaction between 1,4‐butanediol diglycidyl ether and CS. The reaction method was as follows: 0.5 g of CS was added and adequately dissolved in a solution of 4 mL of 15 wt% aqueous NaOH and 9 mL of acetone. Then, 0.3 mL of 1,4‐butanediol diglycidyl ether was added to the mixed solution. The mixture was stirred vigorously and heated to 100 °C for 5 h. After the reaction, hydrochloric acid was used to normalize the pH of the solution to neutral, and crosslinked CS was obtained. The obtained powder was washed thrice with acetone and dried in an oven. The CS hydrogel was obtained by adding 0.4 g of crosslinked CS to 1 mL of normal saline. Similarly, the Mn_3_O_4_@CS hydrogel was obtained by adding 0.1–0.4 g of crosslinked CS and 10–160 µg of Mn_3_O_4_ nanozymes to 1 mL of normal saline.

### Material Characterization

The morphology and crystal structure of the synthesized Mn_3_O_4_ nanozymes were characterized using TEM and HRTEM (JEM‐2100, JEOL, Tokyo, Japan), respectively. Scanning electron microscopy (SEM, S‐4800, Hitachi, Japan) was used to characterize the morphology of the obtained hydrogels. XRD patterns (D8 Advance, Bruker, Ettlingen, Germany) were used to characterize the crystal structures of the obtained nanozymes and hydrogels. The element composition and atomic valence state of the obtained Mn_3_O_4_ nanozymes were analyzed by XPS. FTIR spectra of the obtained nanomaterials and hydrogels were obtained using an IR spectrophotometer (Nicolet Nexus 670, Thermo Fisher Scientific, Inc., Waltham, MA). Elemental distribution and quantification of the hydrogel were performed using an X‐ray electron probe microanalyzer (EPMA).

SOD activity was assayed by a Total Superoxide Dismutase Assay Kit with WST‐8 (#S0101M, Beyotime Biotechnology, Beijing, China). As per the instructions from the reagent vendor, several concentrations of Mn_3_O_4_ (1, 2, 4, 8,16, 32, and 64 ng ul^−1^) and SOD enzyme (#S0086, Beyotime Biotechnology, Beijing, China) (1, 2, 5, 10, 20, 50, and 100 U ml^−1^) were tested in this study. Briefly, WST‐8/enzyme working solution(160 µL), reaction‐starting liquid (20 µL), and sample or control blank (20 µL) were incubated at 37, 4, or 80 °C for 30 min, and the absorbency was assayed by a microplate reader at 450 nm. ΔA450 = A450_control1_− A450_sample_. Inhibition percentage = (A450_control 1_− A450_sample_)/ (A450_control 1_− A450_control 2_) × 100%. In this study, the SOD enzyme (#S0086) was used to test the reliability of the experiment and quantify the activity of Mn_3_O_4_ nanozymes. Moreover, based on these kits, acetic acid and sodium hydroxide were used to change the pH of the reaction solution (a pH of 4 or 10). Similar to the SOD activity, the CAT activity of Mn_3_O_4_ nanozymes was assayed by a catalase assay kit (S0051, Beyotime Biotechnology, Beijing, China). According to the protocol, 250 mM hydrogen peroxide solution was prepared and calibrated by NanoDrop at 240 nm. Next, 250 mM hydrogen peroxide solution (10 µL), catalase detection buffer (blank: 40 µL; catalase standard or sample: 35 µL) and catalase standard or sample (5 µL) were mixed and incubated at 25 °C. After 2 min of incubation, 10 µL of the reaction solution was mixed with 200 µL of color‐developing working fluid and assayed by a microplate reader at 520 nm.

### SW1353 Cell Stimulation by H_2_O_2_

SW1353 cells were cultured in Leibovitz's L‐15 medium with 10% fetal bovine serum (FBS)(ExCell Bio, China). Before stimulation, SW1353 cells were seeded in 12‐well plates and treated with H_2_O_2_ (40 nM) and Mn_3_O_4_ nanozymes (8 µg ml^−1^) to detect the anti‐inflammatory and matrix‐protective effects of Mn_3_O_4_ nanozymes in vitro. SW1353 cells were divided into four groups and cultured and stimulated in 1) phosphate‐buffered saline (PBS), 2) H_2_O_2_, 3) H_2_O_2_ + Mn_3_O_4_ nanozymes, or 4) Mn_3_O_4_ nanozymes for 24 h. The SW1353 cells were then extracted for protein and mRNA quantification.

### Animals

The animals used in this study were wild‐type male mice (BL6J/C57, 10‐week‐old), which were provided by Beijing Vital River Laboratory Animal Technology (Beijing, China) and maintained under pathogen‐free conditions at the Shandong University Qilu Hospital Animal Center. All experimental procedures were approved by the Animal Research Ethics Committee of the Qilu Hospital of Shandong University (#DWLL‐2021‐053).

### The DMM Mouse Model and Treatments

The DMM mouse model was established to confirm the therapeutic effect of Mn_3_O_4_ in vivo, as previously reported. In this study, the mice were divided into four groups (*n* = 6): sham operation, DMM, Mn_3_O_4_@CS treatment, and CS treatment groups. The mice in the sham operation group underwent the same operation, but without ligation. In the DMM group, the mice underwent DMM surgery without any injections. In the Mn_3_O_4_@CS treatment group, the mice underwent DMM operation and were treated with Mn_3_O_4_@CS hydrogel (80 µg ml^−1^, 5 µL) articular injection once a week. In the CS treatment group, the mice underwent the same operation and were injected with CS hydrogel into the articulation once a week. After 4 weeks, the animals were sacrificed. Knee joint tissues from each mouse were collected and labeled for further evaluation.

### Micro‐CT

To analyze articular erosion, the knee joint was fixed for at least 48 h and scanned using a micro‐CT imaging system (Quantum GX2, PerkinElmer).

### Statistical Analysis

The number of replicates and animals is indicated in the figure legends or the Experimental Section. The results are shown as average values ± SD of the mean (S.E.M.). For normally distributed data sets with equal variances, a one‐way analysis of variance (ANOVA) followed by a Tukey post‐hoc test was carried out across groups. Statistical analysis was carried out using GraphPad Prism 8 Software. *p* < 0.05 was considered statistically significant. The significance level was defined as ns (no significance), *p < 0.05, **p < 0.01, ***p < 0.001, and ****p < 0.0001.

## Conflict of Interest

The authors declare no conflict of interest.

## Supporting information

Supporting Information

## Data Availability

Research data are not shared.
