# Supplementary material for: Trimanganese Tetroxide Nanozyme protects Cartilage against Degeneration by Reducing Oxidative Stress in Osteoarthritis
Source: Adv Sci (Weinh). 2023 Apr 23;10(17):2205859. doi: 10.1002/advs.202205859 (PMC10265103; doi:10.1002/advs.202205859)
Supplement: Supplementary file 1 — Supporting Information [file ADVS-10-2205859-s001.pdf]

ADVANCED SCIENCE
Open Access

Supporting Information

for *Adv. Sci.*, DOI 10.1002/advs.202205859

Trimanganese Tetroxide Nanozyme protects Cartilage against Degeneration by Reducing Oxidative Stress in Osteoarthritis

Wenhan Wang, Jiazhi Duan, Wenjun Ma, Bowei Xia, Feng Liu, Ying Kong, Boyan Li, Hang Zhao, Liang Wang, Keyi Li, Yiwei Li, Xiheng Lu, Zhichao Feng, Yuanhua Sang, Gang Li, Hao Xue, Jichuan Qiu* and Hong Liu**

Supporting Information

Trimanganese tetroxide nanozyme protects cartilage against degeneration by reducing oxidative stress in osteoarthritis

Wenhan Wang#, Jiazhi Duan#, Wenjun Ma, Bowei Xia, Feng Liu, Ying Kong, Boyan Li, Hang Zhao, Liang Wang, Keyi Li, Yiwei Li, Xiheng Lu, Zhichao Feng, Yuanhua Sang, Gang Li, Hao Xue*, Jichuan Qiu*, and Hong Liu*

This supporting information includes **Fig. S1-11**, **Materials and Methods** and **Table S1**

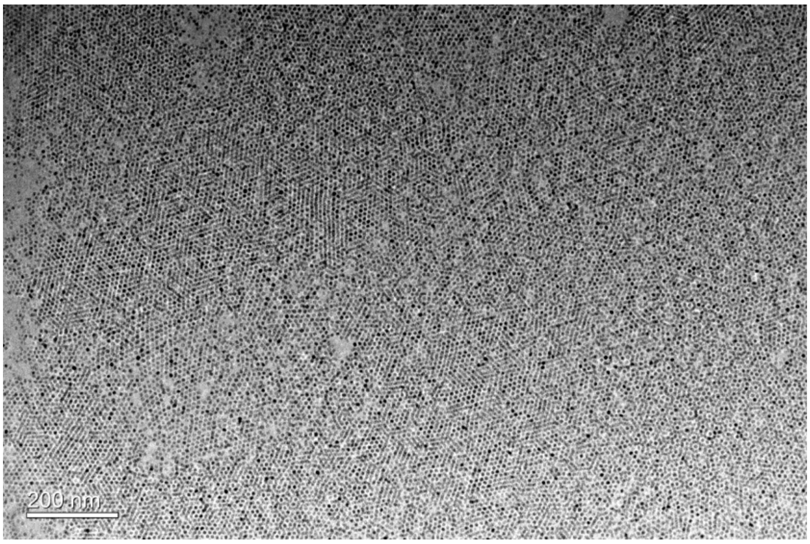

Fig. S1 TEM image of Mn_3O_4 nanoparticles under low magnification.

The TEM image of Mn_3O_4 nanoparticles under low magnification shows that the obtained nanoparticles are of uniform size and the main size distribution are around 6 nm.

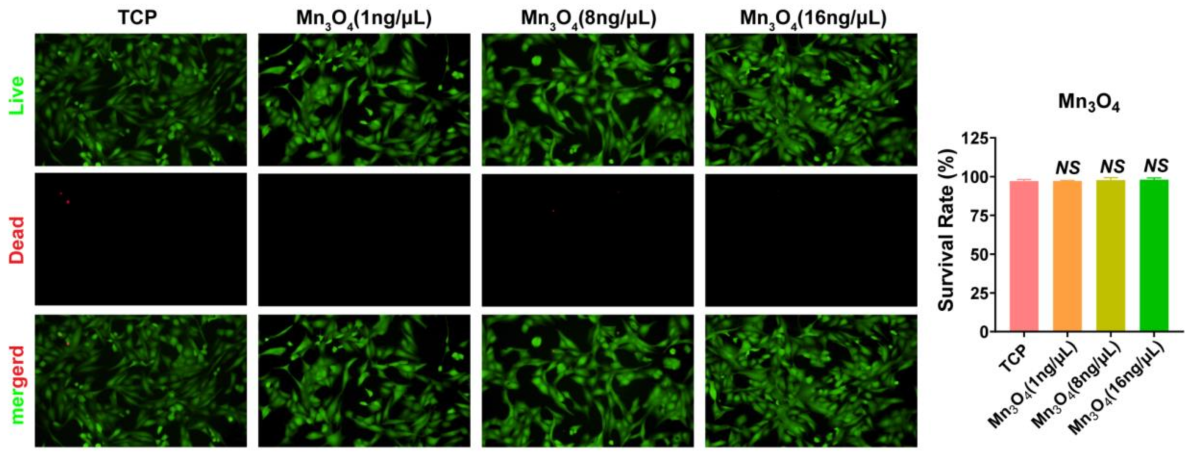

Fig. S2 Live/Dead assay in SW1353 cells of Mn_3O_4 nanoparticles.

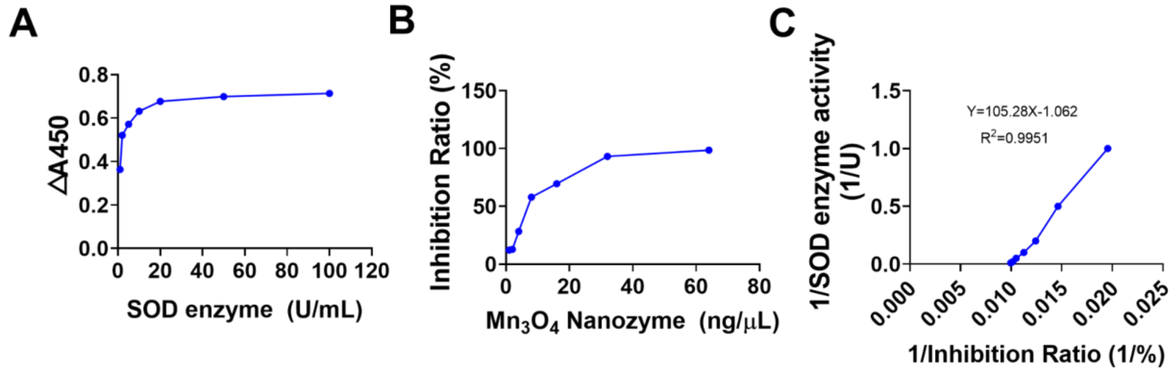

Fig. S3 (**A**) SOD-like activity of SOD bioenzyme. (**B**) Inhibition ratio of various concentration of Mn_3O_4 nanozyme. (**C**) Standard curve of inhibition ratio and SOD activity.

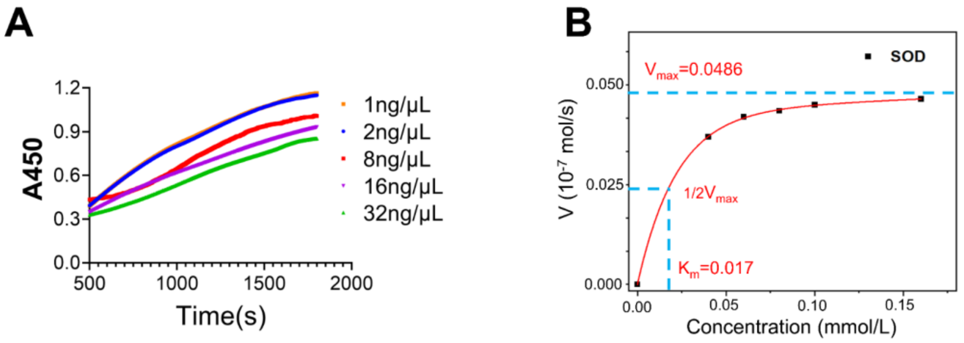

Fig.S4 (A)Kinetic curve of enzymatic reaction of several concentrations (1-32 ng/μL of Mn_3O_4 nanozyme for SOD activity. (B) The concentration of substrate on the SOD catalytic initial velocity of Mn_3O_4 nanozyme.

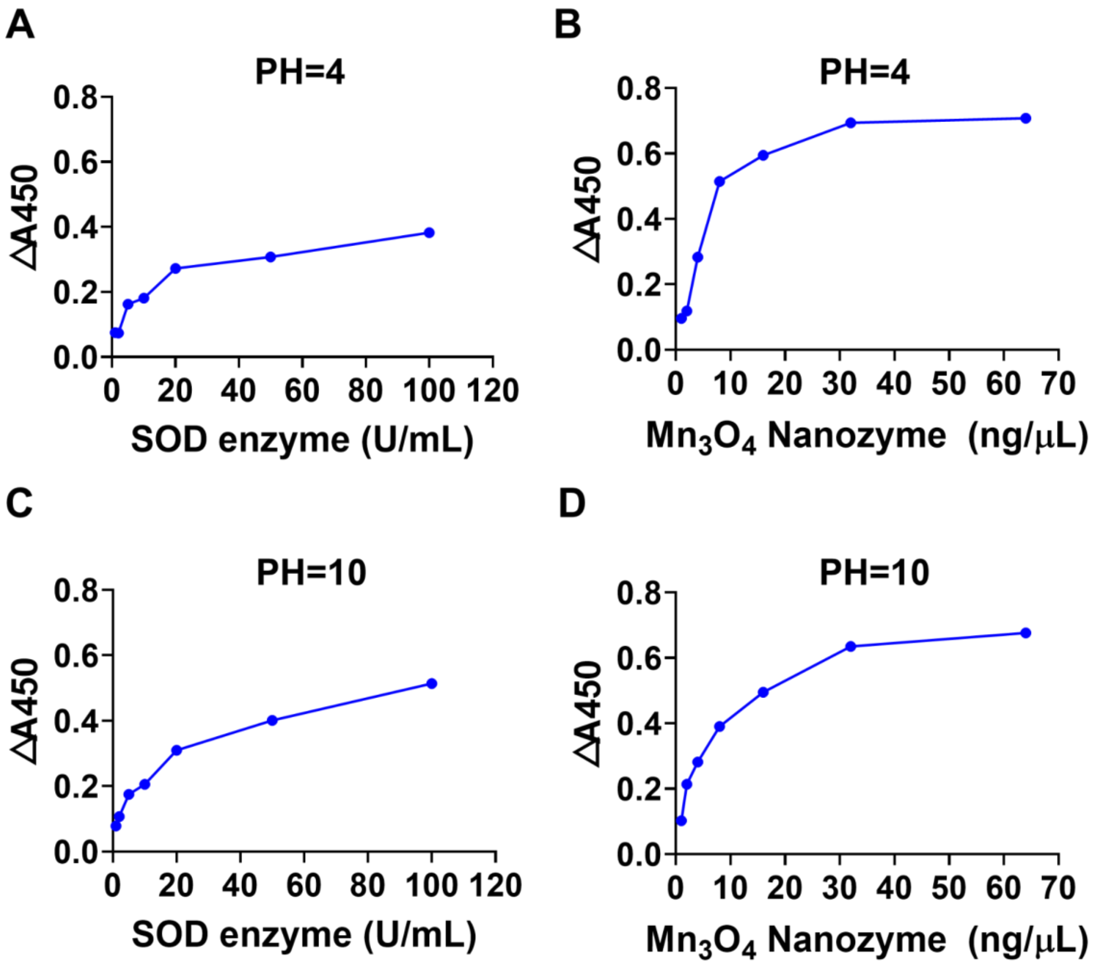

Fig. S5 (**A**)SOD activity of SOD bioenzyme(PH=4). (**B**) SOD activity of Mn_3O_4 nanozyme (PH=4). (**C**)SOD activity of SOD bioenzyme(PH=10). (**B**) SOD activity of Mn_3O_4 nanozyme (PH=10).

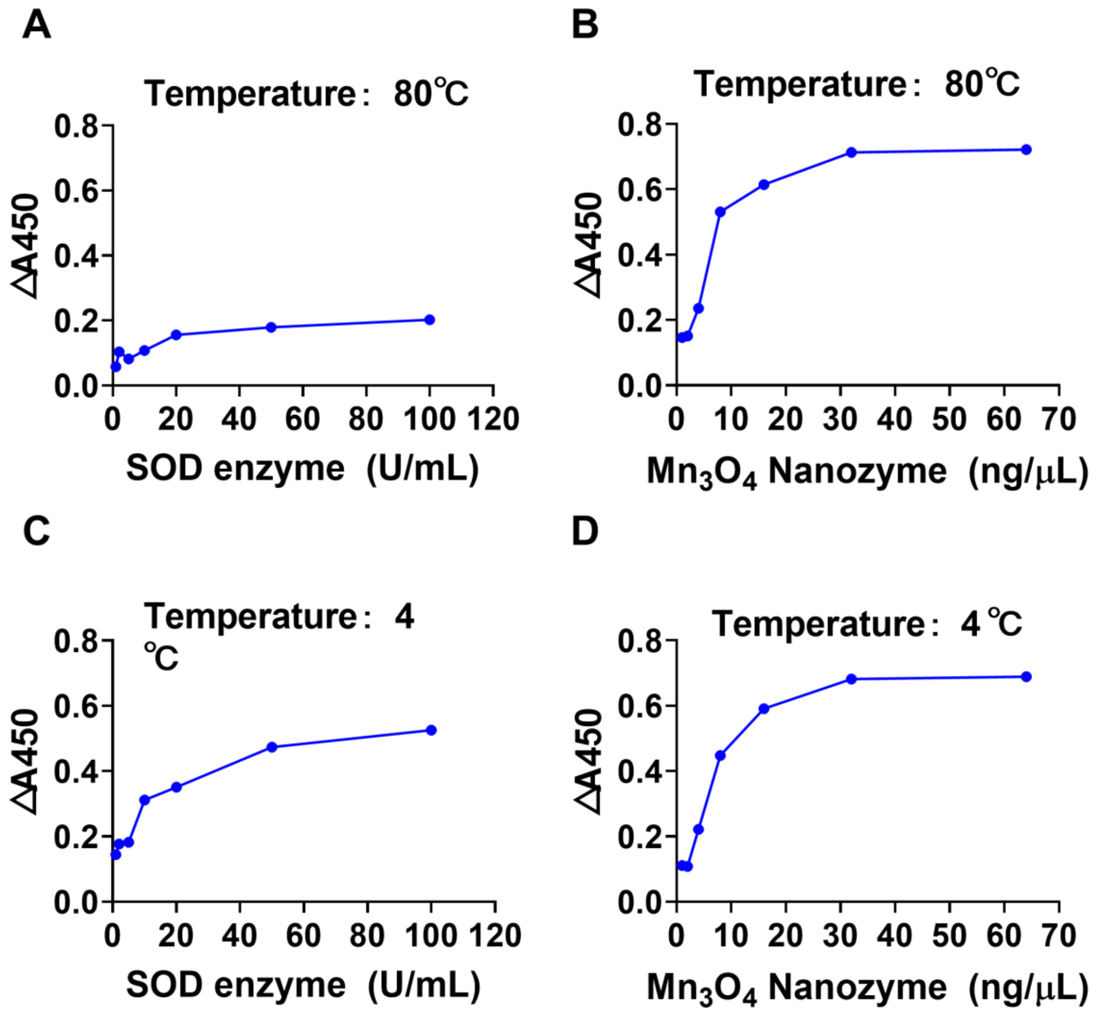

Fig. S6 (**A**)SOD activity of SOD bioenzyme at 80°C. (**B**) SOD activity of Mn_3O_4 nanozyme at 80℃. (**C**)SOD activity of SOD bioenzyme at 4℃. (**B**) SOD activity of Mn_3O_4 nanozyme at 4℃.

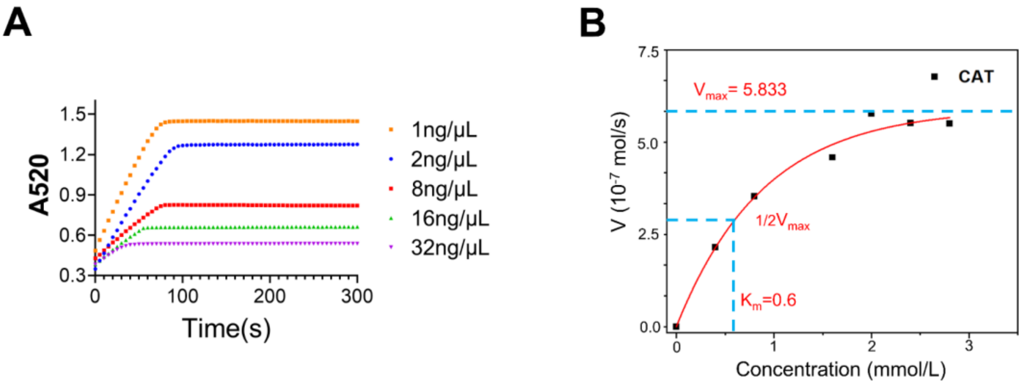

Fig. S7 (A)Kinetic curve of enzymatic reaction of several concentrations (1-32 ng/μL) of Mn_3O_4 nanozyme for CAT activity. (B)The concentration of substrate on the CAT initial velocity of Mn_3O_4 nanozyme.

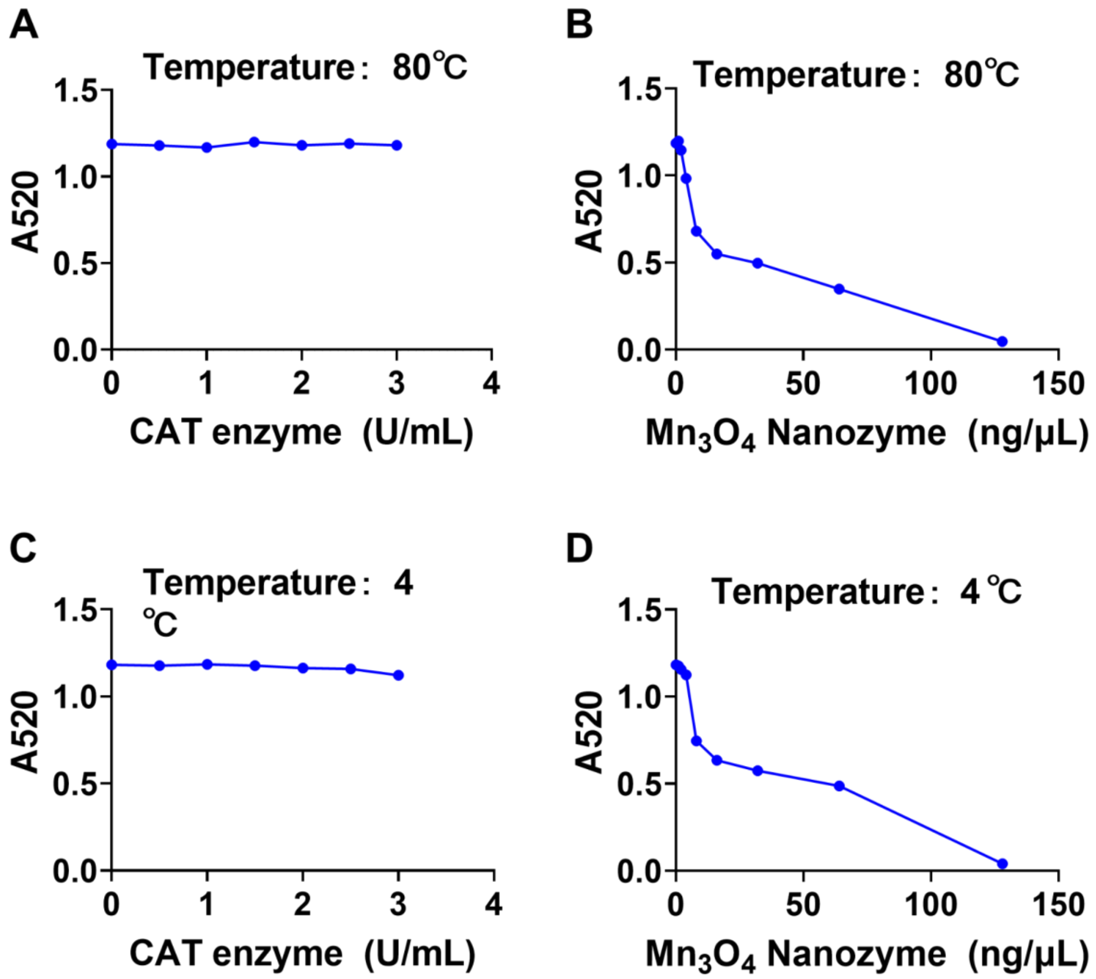

Fig. S8 (**A**)CAT activity of CAT bioenzyme at 80℃. (**B**) CAT activity of Mn_3O_4 nanozyme at 80℃. (**C**)CAT activity of CAT bioenzyme at 4℃. (**B**) CAT activity of Mn_3O_4 nanozyme at 4℃.

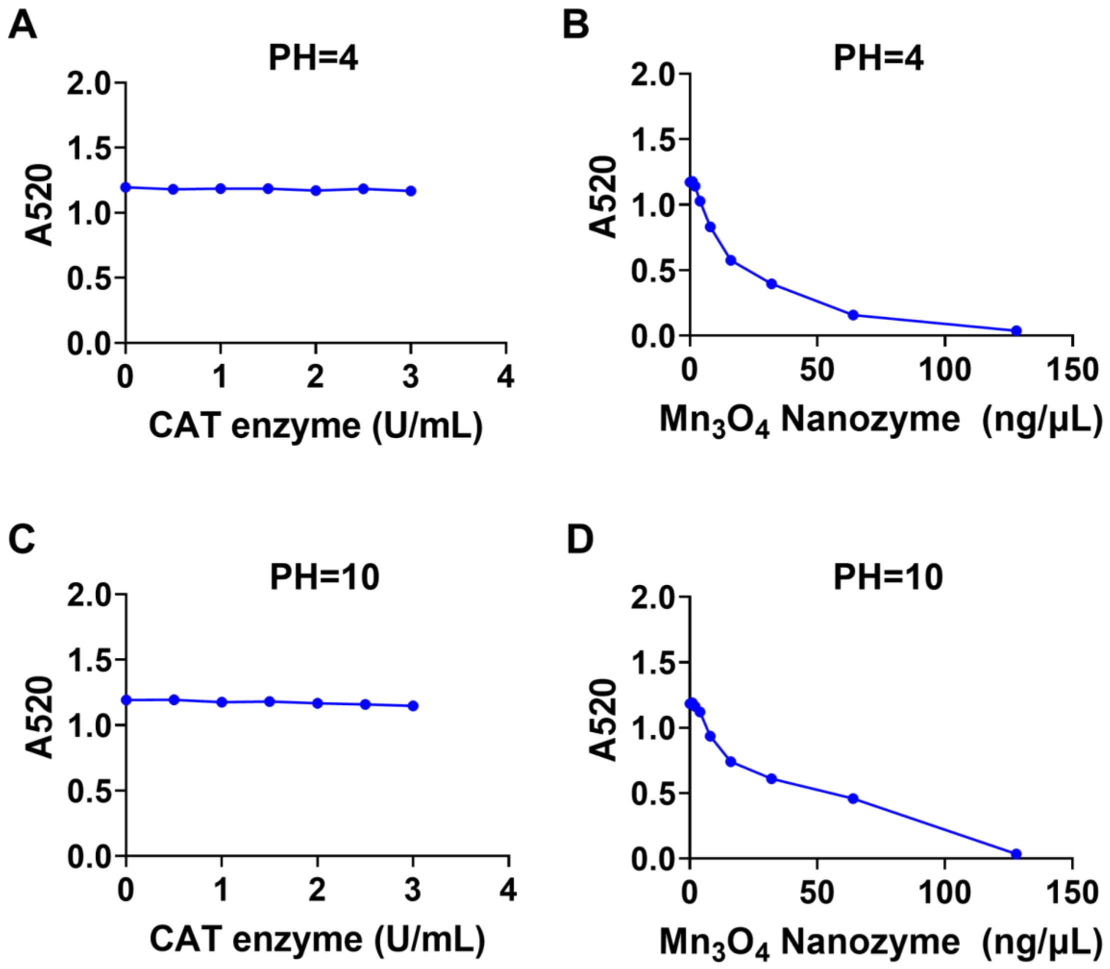

Fig. S9 (**A**)CAT activity of CAT bioenzyme(PH=4). (**B**) CAT activity of Mn3O4 nanozyme (PH=4). (**C**)CAT activity of CAT bioenzyme(PH=10). (**B**) CAT activity of Mn3O4 nanozyme (PH=10).

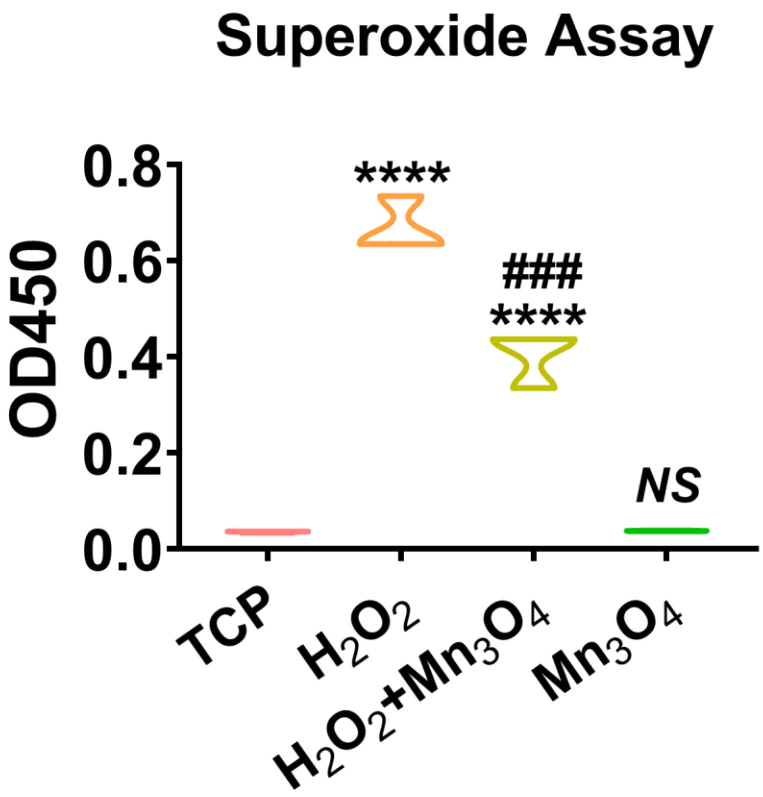

Fig. S10 Superoxide Assay for the four groups.

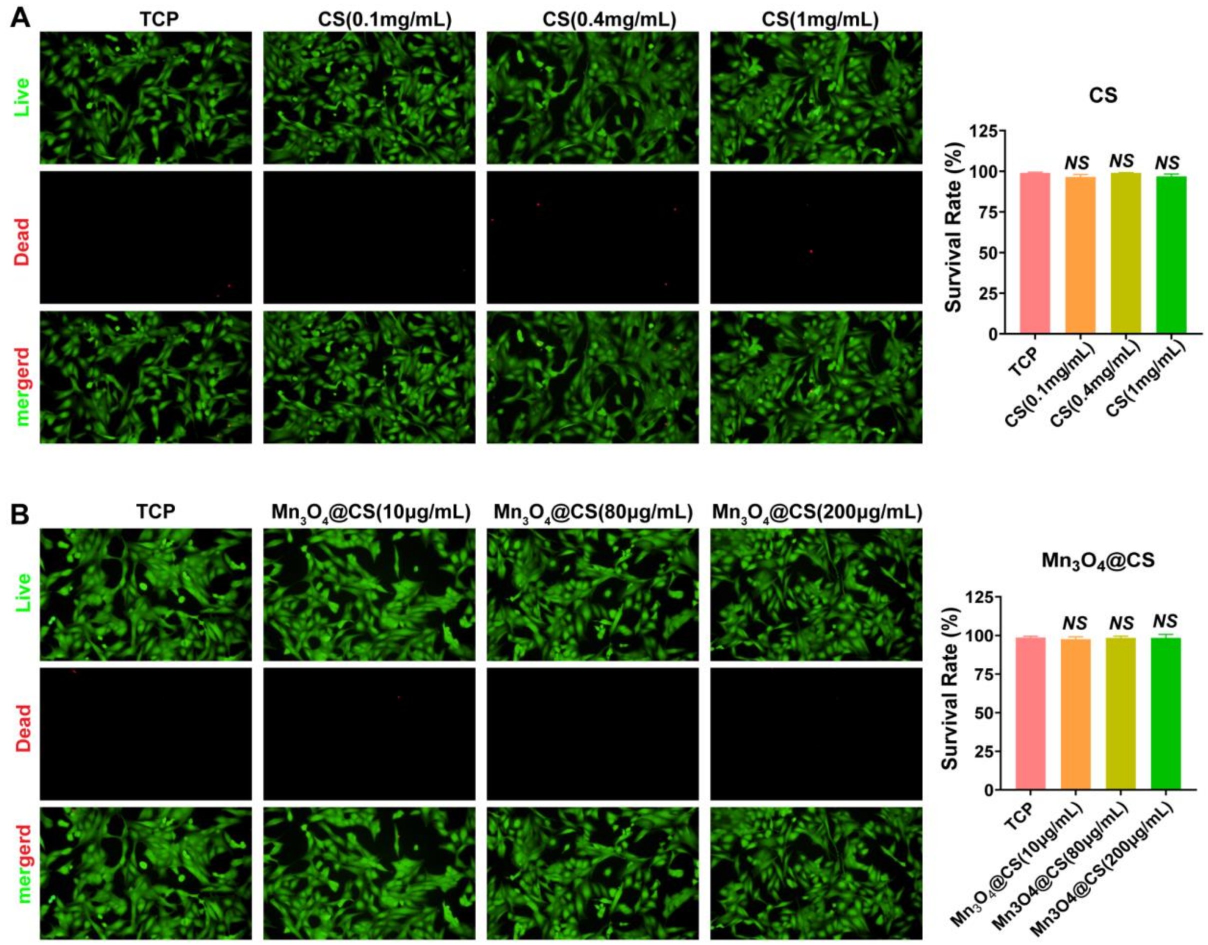

Fig. S11 Live/Dead assay in SW1353 cells of CS hydrogel(**A**) and Mn_3O_4 @CS hydrogel(**B**).

Materials and Methods

Histological and Immunostaining Analysis

Knee joint tissues form each group were fixed in 10% formalin for 72 hours. After decalcification, dehydration and clearance with dimethylbenzene, the Knee joint tissues were embedded in paraffin for the preparation of 5 μm thick sections. For IHC, the sections were incubated with primary antibodies at 4℃ overnight after dewaxing and Antigen repair. Then, we incubated the sections with goat anti-rabbit or anti-mouse IgG horseradish peroxidase HRP for 30 min at 37℃. Finally, immunostaining specificity was evaluated by enhanced polymer detection system kit before dehydration and seal of sections. Besides, the sections were stained with Safranin-O/Fast Green for detecting content of cartilage proteoglycan to reflect the histopathological significance. According to Safranin-O/Fast Green staining, the OARSI scoring system was performed. Briefly, PG depletion was scored as 0–3; complete depletion of PGs (3) to normal (0). Moreover, we scored chondrocyte death on a scale of 0–3; from no empty lacunae (0) to complete loss of chondrocytes (3) in the cartilage layer, and we also scored cartilage surface erosion as 0–3; from no cartilage loss (0) to complete loss of articular cartilage (3). Image J was used to analyze the articular cartilage thickness. Each group contained at least 3 mice, and each mouse was determined and averaged in all sections in 3 parameters.

Intracellular ROS Assay

To explore the intracellular ROS level after Mn_3O_4 treatment, sodium diethyl dithiocarbamate (DDC) and 3-amino-1,2,4-triazole (3-AT) were used to inhibit intercellular SOD and CAT biological enzymes. After DDC+3AT treatment, the cells were treated by H_2O_2 or H_2O_2+Mn_3O_4. Next, the protein was extraceted for SOD or CAT activity assay.

ROS Assay in vivo

To explore the ROS levels in knee joint, Malondialdehyde (MDA) assay kit (S0131S, Beyotime Biotechnology, Beijing, China) and Inhibition and produce superoxide anion assay kit (A052-1-1, Nanjing Jiancheng Bioengineering Institute, Nanjing, China) were used in this study according Manufacturer's instructions. Briefly, knee joints were collected from all groups and total protein were extracted. After concentration determination by BCA test (P0012S, Beyotime Biotechnology, Beijing, China), the total protein were incubated with reaction solution and tested by microplate reader.

Western Blot

Total proteins were extracted and collected from the knee joint tissues and chondrocytes from each treatment group. Protein of each group was resolved on a 10% sodium dodecyl sulfate (SDS) polyacrylamide gel and then transferred by electroblotting to polyvinylidene difluoride (PVDF) membranes. After blocking by 5% milk dissolved in Tris-buffered saline-Tween 20 (TBST) and washed by TBST, the blots in membranes were respectively incubated with COX2 (diluted 1:3000; ProteinTech Group, Chicago, USA), iNOS (diluted 1:3000; ProteinTech Group, Chicago, USA); Aggrecan (diluted 1:1000; Affinity Biosciences LTD, USA); Collagen II (diluted 1:1000; Affinity Biosciences LTD, USA); ADAMTS-5 (diluted 1:1000; Affinity Biosciences LTD, USA); MMP-13 (diluted 1:1000; Affinity Biosciences LTD, USA) at 4℃ overnight. After washing with TBST, all the immunolabeled bands were then incubated with the secondary antibody (1:2000 dilution, ProteinTech Group, Chicago, USA) for 1 h. Finally, we used an enhanced chemiluminescence system (Thermo Scientific, Shanghai, China) to achieve visualization of bound antibody content.

Real-time PCR

Total RNA was collected from the knee joint tissues and chondrocytes from each treatment group by a RNeasy kit (FastPure® Cell/Tissue Total RNA Isolation Kit V2, Vazyme Biotech Co., Ltd, Nanjing, China) in accordance with the instructions of the manufacturer. Then Polymerase Chain Reaction (PCR) kits (AG11728, ACCURATE BIOTECHNOLOGY (HUNAN) CO., Ltd, Changsha, China) were used to reversely transcribe mRNA into cDNA. QPCR was performed with SYBR Green I dye to detect the DNA synthesis. Datas indicated in the experimental groups were analyzed by Light-Cycler analysis software 4.0.0.23 (Roche, Switzerland) after normalization to GAPDH. The sequences of primers used for RT-PCR were synthesized, as shown in **Table S1**. The specific PCR products for each gene were con-firmed by melting-curve analysis. All expression levels of relevant genes in different groups were calculated according to the formula: $2^{-\Delta\Delta Ct}$.

Table S1. The sequences of primers used for RT-PCR

Gene	Species	Sequence (5'→3') F	Sequence (5'→3') R
MMP-13	homo	ATTAAGGAGCATGGCGACTTCT	GCCCAGGAGGAAAAGCATGA
ADAMTS-5	homo	GAAACAACGGACGCTACTGC	ATGATTTACCATTGGGTGGGCA
COL2A1	homo	GATGGCTGCACGAAACATACC	GCCCTATGTCCACACCGAAT
Aggrecan	homo	GGTCTCACTGCCCAACTACC	CACGATGCCTTTCACCACGA
iNOS	homo	TTCAGTATCACAACCTCAGCAAG	TGGACCTGCAAGTTAAAATCCC
COX-2	homo	CTGGCGCTCAGCCATACAG	CGCACTTATACTGGTCAAATCCC
GAPDH	homo	AAATGGTGAAGGTCGGTGTGAAC	CAACAATCTCCACTTTGCCACTG
MMP-13	Mus	TTCTGGTCTTCTGGCACACG	TTGTAGCCTTTGGAACTGCTTG

ADAMTS-5	Mus	TGAGAACTGGATGTGACGGC	GAGTTATCCCCTCCGCACAC
Collagen-2	Mus	CCAACGGCGAGAAGGGAGAA	AGCGAATCCAGCAGGTCCAG
Aggrecan	Mus	CAGATGGCACCCTCCGATAC	GACACACCTCGGAAGCAGAA
iNOS	Mus	GCCACCAACAATGGCAACAT	TCGATGCACAACTGGGTGAA
COX-2	Mus	TGCTGGTGGAAAAACCTCGT	AAAACCCACTTCGCCTCCAA
GAPDH	Mus	AATGGATTTGGACGCATTGGT	TTTGCACTGGTACGTGTTGAT
